# Active symptom control with or without oral vinorelbine in patients with relapsed malignant pleural mesothelioma (VIM): A randomised, phase 2 trial

**DOI:** 10.1016/j.eclinm.2022.101432

**Published:** 2022-05-19

**Authors:** Dean A. Fennell, Catharine Porter, Jason Lester, Sarah Danson, Paul Taylor, Michael Sheaff, Robin M Rudd, Aarti Gaba, Sara Busacca, Lisette Nixon, Georgina Gardner, Liz Darlison, Charlotte Poile, Cathy Richards, Peter-Wells Jordan, Gareth Griffiths, Angela Casbard

**Affiliations:** a Mesothelioma Research Programme, University of Leicester, Robert Kilpatrick Clinical Sciences Building, Leicester LE2 7LX, UK; b University Hospitals of Leicester NHS Trust, Leicester, UK; c Centre for Trials Research, Cardiff University, Wales, UK; d The Rutherford Cancer Centre, Newport, UK; e Sheffield ECMC, University of Sheffield and Sheffield Teaching Hospital NHS Foundation Trust, Sheffield, UK; f Department of Medical Oncology, Wythenshawe Hospital, Manchester University NHS Foundation Trust, Manchester, UK; g Barts Health NHS Trust, London, UK; h The London Clinic, London, UK; i CRUK Southampton Clinical Trials Unit, University of, Southampton, Southampton, UK

**Keywords:** Mesothelioma, Relapsed, Vinorelbine, Pleural, Randomised, BRCA1

## Abstract

**Background:**

Currently, there is no US Food and Drug Administration approved therapy for patients with pleural mesothelioma who have relapsed following platinum-doublet based chemotherapy. Vinorelbine has demonstrated useful clinical activity in mesothelioma, however its efficacy has not been formally evaluated in a randomised setting. BRCA1 expression is required for vinorelbine induced apoptosis in preclinical models. Loss of expression may therefore correlate with vinorelbine resistance.

**Methods:**

In this randomised, phase 2 trial, patients were eligible if they met the following criteria: age ≥ 18 years, Eastern Cooperative Oncology Group (ECOG) performance status 0 or 1, histologically confirmed pleural mesothelioma, post platinum-based chemotherapy, and radiological evidence of disease progression. Consented patients were randomised 2:1 to either active symptom control with oral vinorelbine versus active symptom control (ASC) every 3 weeks until disease progression, unacceptable toxicity or withdrawal at an initial dose of 60 mg/m^2^ increasing to 80 mg/m^2^ post-cycle 1. Randomisation was stratified by histological subtype, white cell count, gender, ECOG performance status and best response during first-line therapy. The study was open label. The primary endpoint was progression-free survival (PFS), measured from randomisation to time of event (or censoring). Analyses were carried out according to intention-to-treat (ITT) principles. Recruitment and trial follow-up are complete. This trial is registered with ClinicalTrials.gov, number NCT02139904.

**Findings:**

Between June 1, 2016 and Oct 31, 2018, we performed a randomised phase 2 trial in 14 hospitals in the United Kingdom. 225 patients were screened for eligibility, of whom 154 were randomly assigned to receive either ASC + vinorelbine (*n* = 98) or ASC (*n* = 56). PFS was significantly longer for ASC+vinorelbine compared with ASC alone; 4.2 months (interquartile range (IQR) 2.2–8.0) versus 2.8 months (IQR 1.4–4.1) for ASC, giving an unadjusted hazard ratio (HR) of 0·60 (80% CI upper limit 0.7, one-sided unadjusted log rank test *p* = 0.002); adjusted HR 0.6 (80% CI upper limit 0.7, one-sided adjusted log rank test *p* < 0.001). BRCA1 did not predict resistance to ASC+vinorelbine. Neutropenia was the most common grades 3, 4 adverse events in the ASC +vinorelbine arm.

**Interpretation:**

Vinorelbine plus ASC confers clinical benefit to patients with relapsed pleural mesothelioma who have progressed following platinum-based doublet chemotherapy.

**Funding:**

This study was funded by Cancer Research UK (grant CRUK A15569).

**Research in context**

**Evidence before this study**

We searched MEDLINE from Jan 1, 2009, to Nov 1, 2021 for clinical trials using the terms “mesothelioma”, “relapsed”, or “pleural”, “phase II”, “randomised”, “vinorelbine” without any language restrictions. This search revealed no evidence of any previously published active symptom controlled, randomised study of vinorelbine monotherapy. Two previously randomised trials (phase III PROMISE-meso study, and a randomised study of the mesothelin antibody drug conjugate anetumab ravtansine) had used vinorelbine as the control arm, but neither met their primary endpoints. Previous single arm phase IIA studies of vinorelbine have shown some clinical activity (level 2b). However, no randomised trial has evaluated vinorelbine versus active symptom control. BRCA1 has been shown in preclinical studies to regulate vinorelbine induced apoptosis, however this has not been prospectively validated in a randomised study.

**Added value of this study**

To our knowledge, VIM is the first active symptom-controlled study of vinorelbine in patients relapsed malignant pleural mesothelioma. VIM met its primary endpoint of progression-free survival and our clinical view is that it demonstrated an acceptable level of safety and tolerability. BRCA1 was not found to be predictive or prognostic in VIM, highlighting the need for other predictors of efficacy for this drug class in mesothelioma.

**Implications of all evidence available**

The lack of an internationally licenced standard of care for patients with relapsed mesothelioma after standard platinum-based chemotherapy, underpinned the design and execution of the VIM trial, with the goal of determining the specific efficacy of the antitubulin agent vinorelbine versus active symptom control in patients with pleural mesothelioma with any histology.

Alt-text: Unlabelled box

## Introduction

Malignant pleural mesothelioma (MPM) is an incurable cancer caused by asbestos. Licenced therapies are lacking for patients following platinum-doublet based chemotherapy.^1^ Single arm phase 2 studies of intravenously administered vinorelbine have reported apparently useful single agent efficacy in patients with relapsed MPM,^2^ leading to its use as a reference control arm in randomised clinical studies. In randomised studies, vinorelbine has been shown to have similar efficacy to monotherapy anti-PD1 checkpoint inhibition by pembrolizumab or mesothelin-targeted antibody drug conjugate.^3^^,^^4^ However, to date there have not been *any* randomised trials designed to confirm whether Active Symptom Control (ASC)+vinorelbine can achieve a meaningful benefit over and above ASC alone.

Vinorelbine causes microtubule depolymerisation, triggering chromosome mis-segregation. This leads to activation of the spindle assembly checkpoint (SAC). We have reported in preclinical studies previously that BRCA1 expression is essential for cell cycle arrest and the induction of apoptosis by vinorelbine.^5^^,^^6^ In pleural mesothelioma, BRCA1 expression is lost in around a third of patients. This suggests that BRCA1 expression may have utility as a predictive biomarker of vinorelbine efficacy.

We report the results of the Vinorelbine in Mesothelioma (VIM) trial, which was designed to evaluate the effectiveness of oral vinorelbine on progression-free survival in patients whose disease had progressed following at least one course of platinum-based chemotherapy.

## Methods

### Study design and participants

This UK-based, multicentre, open label 2:1 randomised trial was designed by the lead authors in collaboration with the sponsor (The University of Leicester). The study protocol was approved by the Wales Research Ethics Committee (14/WA/1054). The study was conducted in accordance with the provisions of the Declaration of Helsinki and Good Clinical practice guidelines as defined by the International Conference on harmonisation. Written informed consent was obtained from all patients prior to enrolment.

Patients who had received at least one course of platinum-based chemotherapy and whose disease had subsequently progressed were deemed eligible for enrolment into the VIM trial. Patients were approached in the hospital setting by research staff. Patients were eligible based on a histologically confirmed diagnosis of pleural mesothelioma with available biopsy material sufficient for determination of BRCA1 expression. Patients had to be ≥ 18 years of age and with an Eastern Cooperative Group (ECOG) performance status of 0–1, and a life expectancy of ≥ 3 months. Patients were required to have adequate bone marrow function with Haemoglobin >100 g/l, white cell count ≥ 3 × 10^9^/L, neutrophil count ≥ 1.5 × 10^9^/L and platelets ≥ 100 × 10^9^/L, and adequate hepatic function with bilirubin < 1.5 x upper limit of normal (ULN) and alanine aminotransferase (ALT) or aspartate aminotransferase (AST) < 2.5 x ULN. Patient's tumours were required to be evaluable as assessed by modified RECIST,^7^ and to have progressed radiologically following pemetrexed/platinum doublet (cisplatin or carboplatin) therapy. Maintenance therapy was allowed following first line treatment (in the context of a clinical trial such as COMMAND^8^ which had enrolled in the UK), and re-challenge with a first-line platinum doublet was also allowed. Accordingly, patients who were enrolled, received oral vinorelbine either as second line or following rechallenge therapy only.

Key exclusion criteria were, known uncontrolled or severe concurrent medical conditions (including brain metastases, severe hepatic insufficiency, long term oxygen requirement), exposure to any live vaccine within the previous 30 days prior to giving consent participate in the clinical trial, or any second malignancy except prostate, cervical cancer in remission, basal cell carcinoma of the skin or superficial bladder cancer. The complete eligibility criteria are provided in the study protocol (Appendix).

### Randomisation and masking

**Figure 1:**
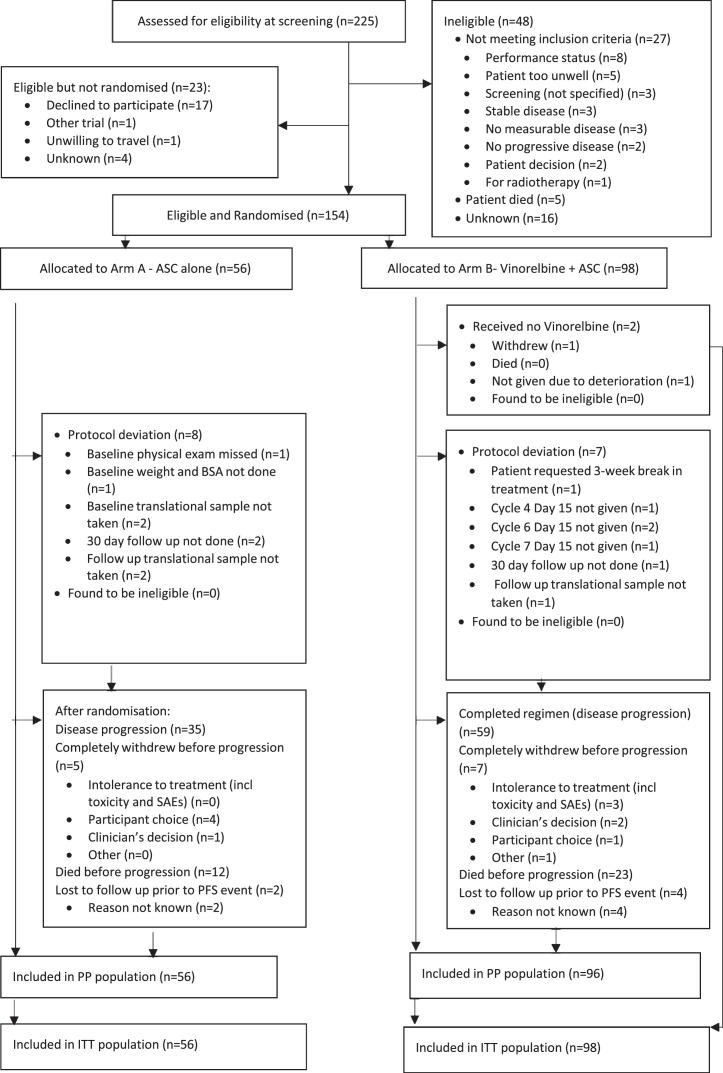
CONSORT flow diagram.

Randomisation (2:1 ASC+vinorelbine vs vinorelbine) was performed centrally by the Centre for Trials Research, Cardiff University and was open-label (non-masked, Figure 1). The randomisation system was developed by and held centrally at the Centre for Trials Research (Wales). The randomisation was stratified using minimisation with a 20% random element. Factors for minimisation included 1st line best response to induction therapy, histology, gender, white cell count and ECOG performance status.

### Procedures

The initial dose of vinorelbine was prescribed at 60 mg/m^2^ orally, on day 1, day 8 and day 15 of a 21 day cycle (ie. weekly), increasing to 80 mg/m^2^ from cycle 2 onwards, until evidence of disease progression, unacceptable toxicity or withdrawal. Patients who reported unacceptable side effects of grade 3 or 4 neutropenia or thrombocytopenia could be dose reduced to 60 mg/m^2^, which was continued until disease progression or unacceptable toxicity. If toxicity returned to ≥ grade 3, vinorelbine was discontinued. Once the dose had been reduced, it could be re-escalated 80 mg/m^2^. Patients were followed up until disease progression, complete withdrawal or death and for 6 months after the end of recruitment.

Patients were assessed by computerised tomography (CT) scans 6 weekly. CT scans were not centrally reviewed. Baseline assessments involved clinical history including assessment of concomitant medications and clinical examination including evaluation of ECOG performance status, CT confirmation of measurable disease according to modified RECIST, and clinical laboratory testing. Weekly assessment of the full blood count was conducted for ASC+vinorelbine only up to 4 days prior to each vinorelbine administration. CT scanning was conducted every 6 weeks.

We previously reported the use of *BRCA1* immunohistochemistry in patients with mesothelioma, validating loss of expression in independent cohorts.^5^^,^^6^^,^^9^ The slides were read by two experts (CR and PW-J). 10% or greater of cells exhibiting medium to strong antibody expression was classed as a positive result.

### Outcomes

The primary endpoint was PFS measured from the time of randomisation until disease progression or death. Outcomes were monitored every 12 weeks following discontinuation of treatment. Progression-free survival was calculated from the date of randomisation to the earliest date of centrally assessed radiological progression (or death from any cause). Radiological progression was defined in the target lesions as at least a 20% increase over baseline. For non-target lesions, unequivocal progression was evaluated as a whole. Patients who were still alive and progression-free at the time of analysis or who dropped out prior to study end were censored at the date of their last modified Response Evaluation Criteria in Solid Tumours (RECIST) assessment. The secondary endpoints were overall survival (OS), objective response rate assessed by modified RECIST,^10^ safety and tolerability. OS was calculated for all patients from the date of randomisation to the date of death (any cause). Patients who were still alive at the time of analysis or who dropped out prior to study end were censored at the day they were last known to be alive. Safety was assessed by evaluating the occurrence of adverse events, which were graded using the National Cancer Institute Common Terminology Criteria for Adverse Events version 4.03. The exploratory research endpoint was correlation of BRCA1 expression with clinical outcome. Research blood samples were taken at baseline and upon disease progression with an optional research re-biopsy conducted using either CT or ultrasound guidance.

### Statistical analysis

The total sample size required to meet the primary endpoint of progression-free survival was 120. VIM was a randomised phase II screening design (hazard ratio (HR) based comparison –log rank test), to explore clinical activity in terms of progression-free survival (PFS) with a HR of 0.65. Using the following parameters; median PFS (control arm) =12 weeks, α= 0.2, β=0.1 (90% power), HR=0.65, one-sided logrank test, 1:2 allocation ratio, 27 month recruitment period, 6 month follow up period, 120 patients, 109 events were required.

The main analysis was intention-to-treat (ITT, all patients allocated to the treatment arm). As a sensitivity analysis, per protocol analyses of PFS were carried out. The main analysis involved a one-sided unadjusted logrank test comparing PFS between the two arms. Median PFS and IQR, and unadjusted HR with 80% CI and one-sided logrank *p*-value were calculated. Analysis of adjusted logrank test and HR was also performed, adjusted for randomisation stratification factors. White cell count has been previously reported as a prognostic factor^11^ and was included as a prognostic factor. PFS and OS were described using Kaplan Meier curves for both arms of the trial. We analysed the data after 109 PFS events. Secondary endpoints were analysed as follows: Median survival and HR for each arm with 95% CI; median PFS and HR for each by BRCA1 status (positive or negative); objective response rate and clinical benefit rate. Descriptive statistics on toxicities and the adherence to the protocol were reported.

### Role of the funding source

The funder of the study, Cancer Research UK had no role in either the trial design, data collection, data analysis, interpretation or authorship. All authors had full access to all the locked data in the study and had final responsibility for the decision to submit for publication.

## Results

Between June 1, 2016 and Oct 31, 2018 we performed a randomised phase 2 trial in 14 hospitals in the United Kingdom. A total of 225 patients were screened and we enrolled 154 patients who were randomised to either ASC (*n* = 56) or ASC+vinorelbine (*n* = 98) (Figure 1). Overall, the most common reason for termination of the study drug was disease progression (94/154; 61%). The demographic and baseline characteristics of the patients were well balanced between arms and are shown in Table 1. Most of the patients were male (125/154; 81%), with a median age of 71 years (ranging from 42 to 83). Baseline ECOG performance score was 0 in 38/154 (25%) and 1 in 115/154 (75%), respectively. Histological subtype was balanced; in the ASC arm [epithelioid (48/56; 86%), biphasic or sarcomatoid (3/56; 5%), and not otherwise specified (NOS) or missing (5/56; 9%)] versus ASC+vinorelbine arm [epithelioid (81/98; 83%), biphasic or sarcomatoid (13/98; 13%) and NOS or missing (4/98; 4%)].

**Table 1:** Patient baseline characteristics, stratification factors and prior therapy.

Characteristics	ASC+Vinorelbine (*N* = 98)	ASC (*N* = 56)
	Median (IQR); *n*	
Age median (IQR)	70.5 (65.4–76.4); 98	70.7 (66.6–74.2); 56
	*n* (%)	
Gender		
Male	80 (82)	45 (80)
Female	18 (18)	11 (20)
ECOG performance status		
0	26 (27)	12 (21)
1	71 (72)	44 (79)
Mesothelioma Subtype		
Epithelioid	81 (83)	48 (86)
Biphasic or sarcomatoid	13 (13)	3 (5)
NOS	3 (3)	5 (9)
Missing	1 (1)	0 (0)
Best response during first line therapy		
DCR (Complete response, partial response or stable disease)	73 (75)	40 (71)
Progressive disease	24 (25)	16 (29)
Missing	1 (1)	0 (0)
Smoking Status		
Smoker	6 (6)	2 (4)
Non-smoker	40 (41)	19 (34)
Ex-smoker	52 (53)	34 (61)
Missing	0 (0)	1 (2)
Asbestos History		
Yes	80 (82)	47 (84)
No	17 (17)	6 (11)
Missing	1 (1)	3 (5)
White cell count (WBC)		
<= 8.2 × 10^9/L	53 (54)	32 (57)
>= 8.3 × 10^9/L	45 (46)	24 (43)

The median duration of exposure to study drug was 2.8 months in the ASC+vinorelbine arm (appendix Table S1). The median number of cycles received was 4 in the ASC+vinorelbine arm. Compliance with vinorelbine administration was 96/98 (98%). Missed doses and dose interruptions occurred in 34/98 (35%), with at least 1 dose reduction and 47/98 (48%) having at least one dose delay in the ASC+vinorelbine arm (appendix Table S1).

**Figure 2:**
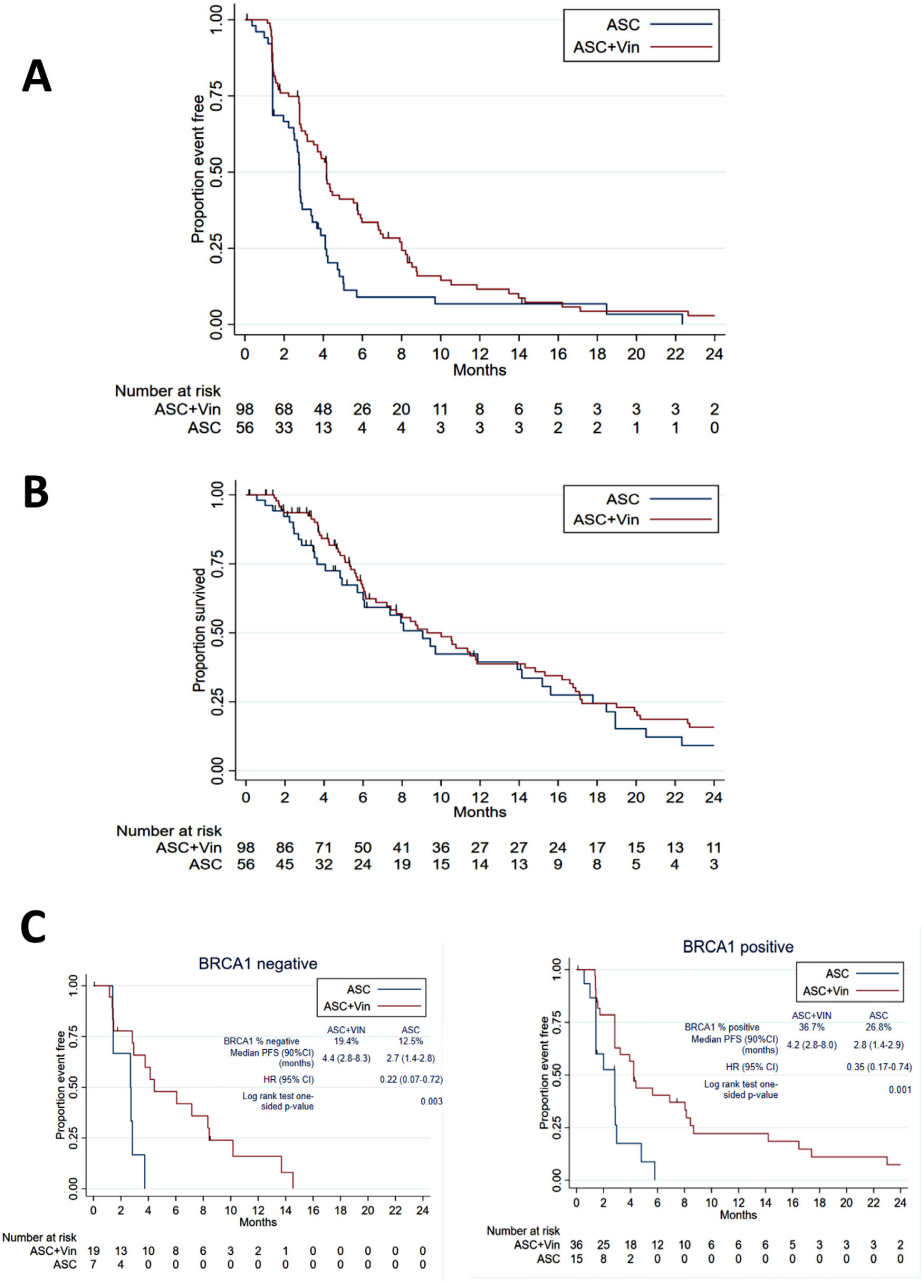
Kaplan-Meier plot. A. Kaplan-Meier plot showing the progression-free survival curves for ASC+vinorelbine versus ASC alone. B. Kaplan-Meier plot showing the overall survival curves for ASC+vinorelbine versus ASC alone. C. Kaplan-Meier plot showing the progression-free survival curves for ASC+vinorelbine versus ASC alone corresponding to (left) BRCA1 negative compared with BRCA1 positive tumour expression.

**Figure 3:**
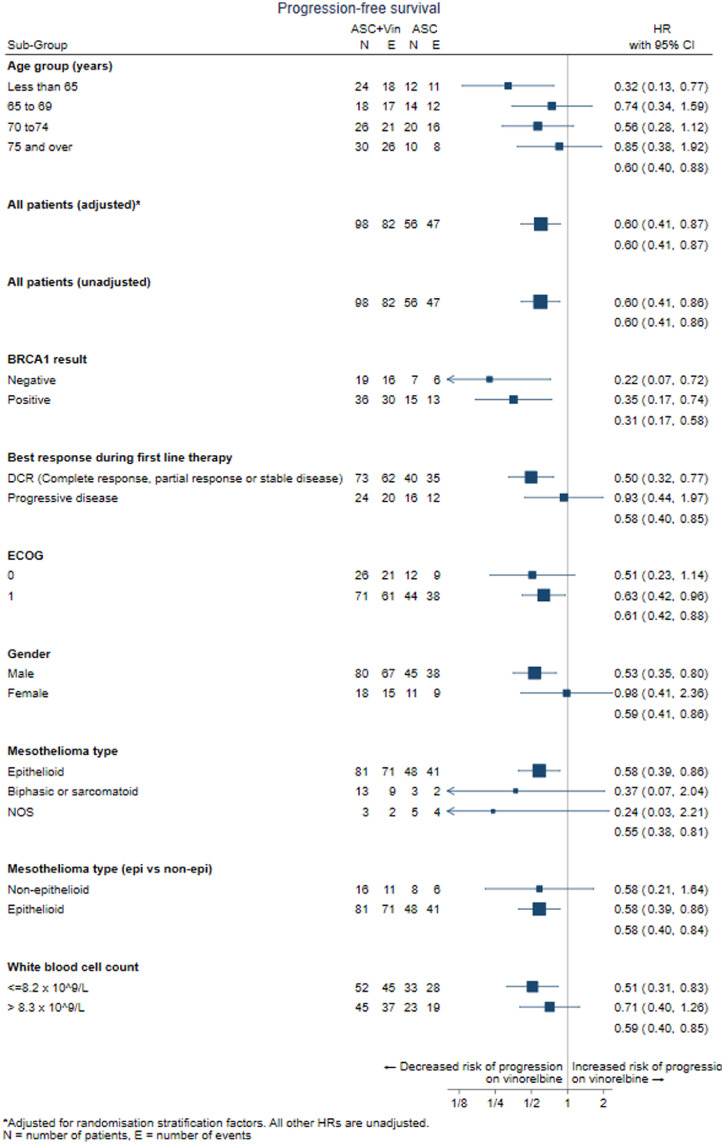
Forest plot of progression-free survival. Progression Free Survival forest plot by subgroups and baseline characteristics (ITT population). *N*=number of patients. *E*=number of events. HR=hazard ratio. *Adjusted for randomisation stratification factors. All other HRs are unadjusted.

Investigator reported median progression-free survival was 4.2 months (IQR 2.2–8.0) for the ASC+vinorelbine arm versus 2.8 months (IQR 1.4–4.1) for ASC; unadjusted one-sided log rank test p-value=0.0022; adjusted one-sided log rank test p-value<0.001,The unadjusted HR was 0.60 (one-sided 80% confidence interval [CI] upper limit 0.7 and adjusted HR 0.6 (one-sided 80% CI upper limit 0.7), corresponding to a significantly longer progression-free survival with ASC+vinorelbine, (Figure 2A). The forest plot of treatment effect (progression-free survival)) in favour of ASC+vinorelbine across sub-groups and baseline characteristics is shown in Figure 3. Disease control achieved during first-line chemotherapy was associated with longer PFS in VIM (Figure 3).

Median overall survival did not differ significantly between treatment arms and was 9.3 months in the ASC+vinorelbine arm versus 9.1 months in the ASC arm (Figure 2B). The best overall response observed was partial response (PR), which was reported in 3 (3%) patients receiving ASC+vinorelbine arm and in 1 (2%) patient in the ASC arm (Table 2). Median duration of PR was 7.2 months (IQR 3.1–8.5) for ASC+vinorelbine compared to 4.2 months (IQR 4.2–4.2) for ASC. Stable disease was observed in 61 (62%) patients in the ASC+vinorelbine arm and 26 (46%) in the ASC arm, corresponding to a disease control rate (i.e. combined partial response + stable disease rates) of 65.3% for ASC+vinorelbine versus 48.2% for ASC (*p* = 0.06).

**Table 2:** Response to ASC+vinorelbine and ASC.

Best Objective Response		ASC+vinorelbine (*N* = 98)	ASC (*N* = 56)
Response (n(%)	Partial response	1 (2)	3 (3)
No response (n(%)	Stable disease	26 (46)	61 (62)
	Progressive disease	16 (29)	19 (19)
	Did not reach Cycle 2 RECIST assessment	7 (13)	8 (8)
	Missing	6 (11)	7 (7)
Median duration of response overall (months)* (95% CIs)		7.2 (3.1–8.5)	4.2 (4.2–4.2)

BRCA1 expression was detected in 51/154 (33.1%) of patients; 26/154 (17%) of patients were BRCA1 negative. This rate is consistent with previously published data^5^^,^^6^; 77/154 (50%) of patients were missing BRCA1 status. Low BRCA1 expression did not predict progression free survival (**see appendix**). vinorelbine was generally well tolerated as shown in Table 3. In both treatment arms, the majority of treatment related adverse events (TRAEs) were mild or moderate in severity. In the per protocol population, the most commonly reported TRAEs of any grade (>20%) in the ASC+vinorelbine arm versus ASC were fatigue (50/96; 52% versus 11/51; 22%); constipation (38/96; 40% versus 4/51; 8%), dyspnoea (31/96; 32% versus 9/51; 18%), diarrhoea (24/96; 25% versus 2/51; 4%), anaemia (23/96; 24% versus 5/51; 10%), nausea (22/96; 23%) vs (2/51; 4%), anorexia (22/96; 23% vs 5/51; 9.8%), and cough (21/96; 22% versus 12/51; 24%). Neutropenia occurred in 18/96 (19%) versus 0%. The most commonly reported Grade 3+ adverse events in the ASC+vinorelbine arm versus ASC were neutropenia (12/96; 13% vs 0/51 (0%), dyspnoea (6/96; 6% vs 0/51; 0%) and lower respiratory infection (5/96; 5% vs 3/51; 6%). 5 patients in the ASC arm had no toxicity data reported.

**Table 3:** Adverse events occurring in >=10% of patients.

	Subjects - N(%) (Grades 1–2)		Subjects - N(%) (Grades 3–5)	
	ASC (*N* = 51)	ASC+vinorelbine (*N* = 96)	ASC (*N* = 51)	ASC+vinorelbine (*N* = 96)
Blood and lymphatic system disorders				
Anaemia	5 (10)	23 (24)	0 (0)	0 (0)
Lymphopenia	2 (4)	12 (12)	0 (0)	4 (4)
Neutropenia	0 (0)	6 (6)	0 (0)	12 (12)
Gastrointestinal disorders				
Abdominal pain	1 (2)	13 (14)	0 (0)	2 (2)
Constipation	4 (8)	37 (39)	0 (0)	1 (1)
Diarrhoea	2 (4)	24 (25)	0 (0)	0 (0)
Nausea	2 (4)	22 (23)	0 (0)	0 (0)
General disorders and administration site conditions				
Fatigue	11 (22)	46 (48)	0 (0)	4 (4)
Chest Pain	5 (10)	12 (12)	0 (0)	2 (2)
Infections and infestations				
Lower respiratory infection	3 (6)	6 (6)	3 (6)	5 (5)
Metabolism and nutrition disorders				
Anorexia	5 (10)	22 (23)	0 (0)	0 (0)
Respiratory, thoracic and mediastinal disorders				
Coughing	12 (24)	21 (22)	0 (0)	0 (0)
Dyspnoea	9 (18)	25 (26)	0 (0)	6 (6)

The incidence of severe adverse events (SAEs) were greater in the ASC+vinorelbine arm than the ASC arm (appendix page 5–7). The most commonly reported SAEs were dyspnoea (5/96; 5% versus 0%), lower respiratory tract infection (5/96; 5% versus 3/51; 6%), unspecified infection (3/96; 3% versus 0%) and febrile neutropenia (3/96; 3%) versus 0%). Grade 5 SAEs causally related to treatment were reported in two (2%) of patients, in the ASC+vinorelbine arm (pneumonia and lower respiratory tract infection). One patient had a fatal SAE in the ASC arm (lower respiratory tract infection).

## Discussion

The VIM trial showed modest but statistically significant (33%) progression-free survival for ASC+vinorelbine versus ASC alone. To our knowledge, VIM is the first positive, active symptom controlled clinical trial of vinorelbine to be reported in patients with relapsed malignant pleural mesothelioma, and adds to the evidence base for effective treatment in this treatment setting. Caution must be drawn regarding the interpretation of efficacy from this randomised phase II study, associated with a 1.4 month improvement in PFS, the use of an alpha (error) of 0.2, lack of central review and given that previous randomised trials in mesothelioma have failed to confirm quite promising phase II signals.^12^^,^^13^ In addition, the higher rate of missing or NOS histology in the ASC+vinorelbine (4%) compared with ASC (9%) could have introduced bias based on the true nature of these histological subtypes. The lack of a double blind placebo controlled design may have influenced the assessment of the efficacy, the safety outcomes, or the ongoing management of participants in the study.

Disease control following either platinum doublet chemotherapy or recently licenced ipilimumab and nivolumab immunotherapy is short at only 6 months.^1^^,^^14^ Following platinum-based doublet therapy, treatment options remain limited with no international consensus on a therapeutic option. Recently, there has been some very promising data reported for switch maintenance gemcitabine, which was associated with a significant improvement in progression-free survival (Hazard ratio 0.48, *p* = 0.0002) following platinum doublet therapy.^15^ VIM was focused on the second line setting, meaning that for many patients now able to receive first line immunotherapy, vinorelbine is now possibly an option post chemotherapy ie. Third line.

In the relapsed treatment setting, two statistically significant randomised trials have been previously reported. The RAMES positive randomised phase II study, compared gemcitabine with or without ramicirumab in the relapsed setting, with evidence of a doubling of PFS and improved overall survival (HR 0.71 *p* = 0.028).^16^ The phase III trial CONFIRM recently reported a significant improvement in progression-free and overall survival in the relapsed setting, comparing nivolumab with placebo (HRs 0.61, *p* = 0.001 and 0.71, *p* = 0.02, respectively).^17^ This was the first phase III trial to show improved survival in the relapsed setting. Together, these studies reflect recent research progress in the relapsed mesothelioma setting which until recently has been at a therapeutic plateau.^18^^,^^19^ The results of VIM provide evidence for an alternative treatment option for these patients. The oral bioavailability of vinorelbine, in contrast to intravenous delivery used in previous trials provides more patient-friendly administration, removing the risk of extravasation injury and weekly infusion requirements.

Previously reported clinical activity for vinorelbine in patients with pleural mesothelioma, can be traced back 2 decades to a UK single arm study which showed a promising response rate of 29%.^20^ The UK randomised phase III trial MS01, although showing no difference overall for MVP/vinorelbine + ASC versus ASC, suggested a longer survival for vinorelbine (HR 0.8, *p* = 0.08), although the study was underpowered to formally test ASC+vinorelbine versus ASC.^21^ The first trial of vinorelbine in the relapsed setting was a single arm phase II study which demonstrated a disease control rate of 68%, ie. comparable with VIM (63%).^2^ Based on this study, many centres around the world have used vinorelbine as a *de facto* standard of care, despite a lack of randomised evidence to justify this. Furthermore, vinorelbine has been used as a control for multiple randomised trials that have failed to demonstrate superiority for experimental treatments, including pembrolizumab^22^ and the antibody drug conjugate aneteumab ravtansine.^23^

In VIM, overall survival was not longer for ASC+vinorelbine compared with ASC. The study was not powered for survival, which may partly explain this. Also, post-study immunotherapy was received by a significant proportion of patients (29%, appendix page 10)due to the concurrent enrolment into the CONFIRM phase III trial and receipt of pembrolizumab, accounting for a likely crossover bias.^17^ Together, these factors may have led to lack of an overall survival signal.

We observed a low objective response rate (3%), highlighting the predominantly cytostatic activity of weekly oral vinorelbine. Review was not centrally performed and the response rate of 2% in the control arm demonstrates the challenges of assessing response in this cancer. The response rate seen in the ASC arm (2%) likely reflects the challenges of assessing response in this cancer. Of note, radiology assessment was not centralised. Previously reported preclinical studies had demonstrated a critical role for BRCA1 in regulating vinorelbine induced apoptosis.^5^^,^^6^ BRCA1 functions via MAD2L1 to mediate activation of the spindle assembly checkpoint in response to vinorelbine, and loss of expression via siRNA mediated silencing, reproducible blocks the induction of cell death induced by this drug.^5^ However, in VIM, loss of expression of BRCA1 which is only very rarely mutated in mesothelioma,^24^ was not found to be correlated with clinical activity.

BRCA1 assessment was only possible in 50% if the trial population, being a significant limitation of this study. One reason for this is that low (<10%) but not absent BRCA1 expression may be sufficient to mediate spindle assembly checkpoint activity in response to vinorelbine. Interestingly, prior response to first line chemotherapy was associated with clinical benefit in VIM, implicating a common cross-resistance mechanism (unplanned post-hoc analysis). Both epithelioid and non-epithelioid patients benefitted, however due to the small sample size of the non-epithelioid population, the benefit in favour of ASC+vinorelbine was not significant (unplanned, post-hoc analysis).

Vinorelbine exhibited an acceptable toxicity profile overall which may relate to the relatively low-dose weekly scheduling which was in general tolerable in patients with ECOG performance status 0-1. Although quality of life data could have provided more information regarding clinical benefit, this was not collected in this phase II trial. Dose escalation to 80 mg/m2 at cycle 2 day 1 as per protocol was tolerated in the majority of patients, with 28% requiring a single dose reduction, and only 7% requiring two or more reductions. In view of the modest treatment benefit conferred by vinorelbine, both quality of life and cost effectiveness could be considered important information that were not collected in this study.

In conclusion, the VIM trial showed modest but statistically significant activity in patients with relapsed malignant mesothelioma, with an acceptable safety profile compared with active symptom control. BRCA1 expression does not predict progression-free survival, and therefore further studies are required to understand the factors which underpin sensitivity to this agent. VIM provides important evidence to justify its use as a control arm in future randomised controlled clinical trials.

## Contributors

DAF, GG (Griffiths), GGr (Gardiner), LN, and JL were responsible for study conceptualisation and study acquisition. DAF, AC, and CP were responsible for methodology, formal analysis, and writing the original draft. CP and AC were responsible for formal analysis, validation and verification of underlying data. GGr and LN were responsible for project management. RR, SD participated in the trial management group. JL, SD, PT, LD were investigators responsible for trial recruitment. PWJ,CP,MS, SB, CR, and AG were responsible for biomarker development, data curation, and investigation

All authors were responsible for writing, reviewing and editing, had full access to all the locked data in the study and accept responsibility to submit for publication.

## Data sharing

Trial data relating to this publication shall remain confidential to the sponsor organisation and will not be disclosed, except where disclosure might be required in accordance with pharmacovigilance duties of the parties involved. Individual participant data can be made available, after deidentification, to investigators who provide a written request in accordance with General Data Protection Regulation and following authorisation from the sponsor organisation, starting immediately and ending 3 years after publication. Data sharing requests should be directed to DAF and A.C. The Centre for Trials Research (Cardiff) is committed to the responsible sharing of clinical trial data and trial samples with the wider research community. Data access is administered through the Centre for Trials Research Data Release Committee. Requests for data access and sharing trials should be completed onto the request for data form and emailed to the Chief Investigator (DAF).

## Funding

This study was funded by Cancer Research UK (grant CRUK A15569).

## Declaration of interests

Prof Fennell reports grants from Astex Therapeutics, Boehringer Ingelheim, MSD, and Bayer; personal fees from Aldeyra, Inventiva, and Paredox; non-financial support from Clovis, Eli Lilly, and BMS; and personal fees and non-financial support from Roche, during the conduct of the study. Prof Griffiths reports grants from Jannsen-cilag, grants and personal fees from AZ, grants from Novartis, grants from Astex, grants from Roche, grants from Heartflow, personal fees from Celldex, grants from BMS, grants from BioNtech, outside the submitted work. All other authors declare no competing interests.
